# Targeting signaling pathways in neurodegenerative diseases: Quercetin's cellular and molecular mechanisms for neuroprotection

**DOI:** 10.1002/ame2.12551

**Published:** 2025-01-22

**Authors:** Md. Rezaul Islam, Md. Ibrahim Khalil Al‐Imran, Mehrukh Zehravi, Sherouk Hussein Sweilam, Mohammad Rakib Mortuza, Jeetendra Kumar Gupta, Thukani Sathanantham Shanmugarajan, Kadirvel Devi, Tanuja Tummala, Mohammed Ali Alshehri, Kalirajan Rajagopal, Mohammed Asiri, Irfan Ahmad, Talha Bin Emran

**Affiliations:** ^1^ Department of Pharmacy, Faculty of Health and Life Sciences Daffodil International University Daffodil Smart City Bangladesh; ^2^ Department of Clinical Pharmacy College of Dentistry and Pharmacy, Buraydah Private Colleges Buraydah Saudi Arabia; ^3^ Department of Pharmacognosy College of Pharmacy, Prince Sattam Bin Abdulaziz University Al‐Kharj Saudi Arabia; ^4^ Department of Pharmacognosy, Faculty of Pharmacy Egyptian Russian University Cairo Egypt; ^5^ Department of Chemistry and Biochemistry Lamar University Beaumont Texas USA; ^6^ Department of Pharmacology Institute of Pharmaceutical Research, GLA University Mathura India; ^7^ Department of Pharmaceutics, School of Pharmaceutical Sciences Vels Institute of Science, Technology and Advanced Studies (VISTAS) Chennai India; ^8^ Department of Polymer Chemistry Pittsburg State University Pittsburg Kansas USA; ^9^ Department of Biology, Faculty of Science University of Tabuk Tabuk Saudi Arabia; ^10^ Department of Pharmaceutical Chemistry JSS College of Pharmacy, JSS Academy of Higher Education and Research Ooty India; ^11^ Department of Clinical Laboratory Sciences College of Applied Medical Science, King Khalid University Abha Saudi Arabia

**Keywords:** neurodegeneration, neurodegenerative diseases, neuroprotection, quercetin, signaling pathways, clinical studies

## Abstract

**Background:**

Neurodegenerative diseases (NDs), including Alzheimer‘s disease, Parkinson‘s disease, and Huntington‘s disease, are complex and challenging due to their intricate pathophysiology and limited treatment options.

**Methods:**

This review systematically sourced articles related to neurodegenerative diseases, neurodegeneration, quercetin, and clinical studies from primary medical databases, including Scopus, PubMed, and Web of Science.

**Results:**

Recent studies have included quercetin to impact the cellular and molecular pathways involved in neurodegeneration. Quercetin, a flavonoid abundant in vegetables and fruits, is gaining attention for its antioxidant, anti‐inflammatory, and antiapoptotic properties. It regulates signaling pathways such as nuclear factor‐κB (NF‐κB), sirtuins, and phosphatidylinositol 3‐kinase/protein kinase B (PI3K/Akt). These pathways are essential for cellular survival, inflammation regulation, and apoptosis. Preclinical and clinical studies have shown that quercetin improves symptoms and pathology in neurodegenerative models, indicating promising outcomes.

**Conclusions:**

The study explores the potential of incorporating laboratory research into practical medical treatment, focusing on quercetin‘s neuroprotective effects on NDs and its optimal dosage.

## Graphical abstract

Quercetin regulates signaling pathways in neurodegenerative diseases, including NF‐κB, sirtuins, and PI3K/Akt. Studies show quercetin improves symptoms and pathology in neurodegenerative models. The study aims to incorporate laboratory research into practical medical treatment, focusing on quercetin's neuroprotective effects and optimal dosage.

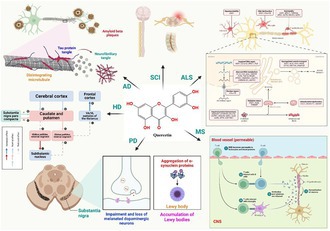

## 1 INTRODUCTION

Neurodegenerative diseases (NDs) are a wide variety of disorders characterized by progressive and moderate death of neuronal cells.^1^ Increasing oxidative stress (OS) is the typical reason for several NDs.^2^ Several risk factors, such as Alzheimer's disease (AD) and Parkinson's disease (PD), are related to the development of NDs.^3^ PD is the second most frequent ND after AD, which impacts 10%–50% of the older population.^3^ NDs are a significant threat to life and health care. Nutritional therapies, primarily based on polyphenols, are increasingly used to prevent these diseases. Quercetin, a common flavonoid with numerous health benefits, is the major component of these therapies. The primary risk factor for ND is aging. The main factors that lead to neurodegeneration include OS and malfunction in the mitochondria. The increase in NDs is concerning due to the limited availability of effective treatment options. Recent research indicates quercetin's potential to improve brain health through various mechanisms, including enhanced cognitive function.^4^ Up to 2020, 18 896 people in India were reported to have PD. The incidence of PD was 42.3 persons per 0.1 million overall; however, this increased with age, with those over 60 having a PD prevalence of 308.9 persons per 0.1 million.^5^ By 2021, there were approximately five to eight Huntington's disease (HD) diagnoses worldwide per 0.1 million people.^6^ However, estimates suggest that by 2050, the life expectancy gap between sub‐Saharan Africa and the global average will decrease to just 10 years. By then, 7.6% of the population (expected to be 2.074 billion) will be ≥60 years. This represents ~156.7 million individuals, or four times the 2010 estimates, in total numbers.^7^ The antioxidant of quercetin capacity has been investigated at the level of brain tissue. The aging process of neurons is linked to the start of the neurodegenerative process. Amyloid beta (Aβ) plaques, Pick bodies, Lewy bodies, neurofibrillary tangles, and other formations can be seen in various brain regions, and they can cause AD, PD, HD, and amyotrophic lateral sclerosis (ALS).^8^ The ability of quercetin to scavenge free radicals has been studied. It protects against OS‐induced neuronal damage by regulating the expression of nuclear factor erythroid 2‐related factor 2 (*Nrf2*)‐dependent antioxidant‐responsive elements and reducing neuroinflammation by inhibiting the NF‐κB signal transducer and STAT1. Quercetin has been demonstrated in several in vitro and in vivo experiments to destabilize and improve the removal of aberrant proteins, including hyperphosphorylated tau and Aβ peptide, which are important pathology indicators of AD. By modifying a variety of kinase signaling cascades, including protein kinase C (PKC), protein kinase B (AKT/PKB) tyrosine kinase, and phosphatidylinositol 3‐kinase (P13‐k), quercetin promotes neurogenesis and prolongs the life of neurons. It improves memory and reverses cognitive decline as people age.^9^ The major mechanisms responsible for quercetin's neuroprotective effects include its suppression of Quercetin's neuroprotective effects involve suppressing polyglutamine aggregation, acetylcholinesterase (AChE), fibrillogenesis of Aβ, 6‐hydroxydo‐pamine (6‐OHDA), 3‐nitropropionic acid (3‐NPA), and elevation of apolipoprotein E (ApoE) levels.^10^, ^11^ Quercetin can help prevent and treat NDs such as AD and PD. OS, a by‐product of energy uptake and metabolism, is a significant mediator of cell death and NDs. Quercetin can reduce inflammation and OS, making it a powerful agent against NDs. However, biological manipulation of Nrf2/HO1 signaling pathways may be a potential therapeutic strategy.^12^ The review describes quercetin's various neuroprotective mechanisms for combating NDs. It can potentially be therapeutic for neurodegenerative patients by targeting OS, inflammation, apoptosis, and neurotrophic deficiencies. The full therapeutic potential of quercetin in NDs requires further understanding of its signaling pathways and clinical trials.

## 2 ABSORPTION, METABOLISM, AND BIOAVAILABILITY OF QUERCETIN

Quercetin glycosides such as quercetin arabinoside are deglycosylated to form quercetin aglycone before being inactively absorbed in the small intestine.^13^ Studies on rats and pigs show quercetin distribution in the lung, liver, colon, kidney, and brain, with reduced amounts in the brain.^14^ Dietary quercetin can be increased to low micromolar levels by supplementing with quercetin aglycone or glycosides.^15^, ^16^ Quercetin can significantly increase plasma concentrations when supplemented.^16^, ^17^ After quercetin consumption, only trace levels of quercetin aglycone are detected.^18^ Research has demonstrated the antioxidant properties of glucuronidated metabolites in vivo and in vitro.^19^, ^20^ Methylation and sulfate metabolites have been linked to additional biological effects,^21^, ^22^ but no evidence has been found for quercetin metabolites.^23^ The conjugated quercetin can enter an erythrocyte and change into its nonconjugated form, which is equally significant.^24^ Quercetin, a substance found in the brain, has the potential to cross the blood‐brain barrier (BBB), influencing its concentration in neural tissue for in vivo application.^25^, ^26^ Brain tissue in rats and pigs administered quercetin in vivo shows modest levels of the compound.^27^, ^28^ Specifically, the absorption of quercetin into the brain is much enhanced when it is formulated in lipid nanoparticles.^29^, ^30^ Furthermore, it has been demonstrated that coadministration of α‐tocopherol with quercetin increases the transport of quercetin across the BBB.^31^ The high lipophilicity of quercetin leads to its low solubility in water and subsequent bioavailability after intake. Quercetin is available in small amounts for peripheral tissues and organs. Rats were shown the absolute bioavailability of 16% in aqueous solution and 27.5% in ethanol‐polyethylene glycol.^32^, ^33^ Glycosides make up the majority of dietary flavonoids. Before being absorbed, β‐glucosidases hydrolyze glycosides in the gastrointestinal system to the aglycon. They are then changed into methylated derivatives or conjugates of glucuronide and sulfate. The primary form of quercetin discovered within onions is called isoquercitrin. The absorption of quercetin is higher in the circulation than unglycosylated, unadulterated quercetin or its other glycosides, such as galactoside or rutinoside (rutin).^32^ Quercetin metabolites could pass through the BBB and accumulate within the cerebral tissue to have pharmacological effects.^27^

## 3 NEUROINFLAMMATION TOXICITY AND NEUROPROTECTION

When the immune system responds to damage or disease in the neurological system, it causes neuroinflammation.^34^ Neuroinflammation may not always be the cause of the initial injury. It is a significant factor in the onset and progression of numerous brain damage. Inflammatory processes in the brain can lead to neuronal damage, abnormal brain function, and progression of these disorders.^35^, ^36^ Neuroprotection strategies aim to protect the nervous system from harm or disease by reducing inflammation, promoting neuronal survival and development, or eliminating harmful compounds from the brain.^37^ Tauroursodeoxycholic acid (TUDCA) treatment inhibits the harmful effects of 1‐methyl‐4‐phenyl‐1,2,3,6‐tetrahydropyridine (MPTP) in mouse brains, maintains antioxidant enzyme levels, and upregulates Annexin A1 (ANXA1) expression, thereby negatively influencing neuroinflammation. This is linked to neuroprotection, as demonstrated in microglial models.^38^

## 4 EFFICACY OF QUERCETIN IN VARIOUS NEURODEGENERATIVE DISEASES

Quercetin, a flavonoid with neuroprotective properties, is being explored for its potential use in treating NDs like AD, PD, HD, multiple sclerosis (MS), and spinal cord injury (SCI) (Table 1).

**TABLE 1:** Quercetin exhibits neuroprotective properties against neurodegenerative diseases in various studies.

Disease name	Study model	Dose/concentration	Findings	Reference
Alzheimer's disease	Mice	100 mg/kg	Reduced the onset of cognitive decline and histopathological features in AD	[39]
	Mice	2 mg/g	Quercetin's impact on brain levels of quercetin and the regulation of genes related to AD and antioxidants	[28]
	Mice	40 mg/kg	Enhanced cognitive functioning in AD mouse	[40]
	Mice	25 mg/kg	These nanoparticles enhance the oral absorption of quercetin, indicating their potential as a therapeutic tool in AD pathogenesis	[41]
	Mice	25 mg/kg	Improved the histological signs of AD in aged 3xTg‐AD mice	[42]
	PC‐12 cells	0, 10, 20, 40, and 80 μmol/L	Enhanced PC12 survival rate, promoted cell proliferation, and counteracted Aβ_25‐35_ toxicity	[43]
	Wistar rats	40 and 80 mg/kg	Determination of quercetin on the acquisition and retention of spatial memory AD	[44]
	Mice	–	Quercetin can affect memory recall	[45]
	Rats	25 and 50 mg/kg	Activated the non‐amyloidogenic pathway in a rat model of AD	[46]
	Male Sprague–Dawley rats	100 mg/kg	Quercetin and sitagliptin combination activated Nrf2 signaling against Aβ‐induced AD in rats	[47]
	Wistar rats	80 mg/kg	Regular exercise and quercetin enhance spatial memory and reduce OS markers in AD	[48]
	Male Wistar rats	0.5 mg	Nasal administration of quercetin liposomes enhances cognitive function and protects against AD	[49]
	Male Wistar rats	–	Quercetin‐conjugated superparamagnetic iron oxide nanoparticles protect against AlCl_3_‐induced neurotoxicity in AD	[50]
	Male Wistar rats	50 mg/kg	Quercetin has properties targeting AD‐related genes and slowing cognitive impairment progression	[51]
	Male Wistar rats	50 mg/kg	Improved cholinergic and dopaminergic dysfunctions by lowering acetylcholinesterase levels	[52]
Parkinson's disease	Male Wistar rats	100, 200, 300 mg/kg	Quercetin's cognitive enhancement is attributed to its ability to reduce oxidative damage	[53]
	Rats	50 mg/kg	Enhanced autophagy in PD rat models alters the microenvironment that triggers neuronal death	[54]
	Rats	20 μM	Protected neurons in PD models	[55]
	PC12 cells	–	Rutin and isoquercitrin impact pretreatment on gene expression changes in rat PC12 cells	[56]
	Rats	25–75 mg/kg	Repaired mitochondrial electron transport defects and regulated neuroprotective mechanisms in mitochondrial neurotoxin‐induced parkinsonism	[57]
	Mice	50, 100, and 200 mg/kg	Improved dopamine depletion in brain tissue induced by MPTP treatment	[58]
	Rats	10 and 25 mg/kg	Quercetin's antioxidant properties in the hippocampal regions may be responsible for its cognitive improvement	[59]
	Male Sprague–Dawley rats	–	Evaluated the protective capacity of quercetin nanosomes in an experimental model of PD in rats	[60]
	Male Sprague–Dawley rats	20 mg/kg	Quercetin has neuroprotective properties in the 6‐OHDA model of PD	[61]
	Rats	25 mg/kg	Fish oil and quercetin improve neuroprotection in PD	[62]
	Male Sprague–Dawley rats	30 mg/kg	Mitigated OS in the striatum and reduced dopaminergic neuronal loss in a rat model of PD	[63]
	Rats	–	Quercetin impacts PD	[64]
	PC12 cells	12.5, 25, 50, 100, and 200 μM	Quercetin has a neuroprotective effect on PD treatment	[65]
	Rats	10–100 μM	Prevented oxygen radical formation, cytotoxicity, and neurotoxicity induced by 6‐OHDA	[66]
	Rats	25–100 mg/kg	Affected the levodopa–carbidopa combination in rats, specifically against perphenazine and reserpine‐induced catalepsy	[67]
	Rats	25 mg/kg	The combination of piperine and quercetin effectively prevents the neurotoxicity of 6‐OHDA in rats	[68]
Huntington's disease	Male Wistar rats	50 mg/kg	The combination of lycopene and quercetin reduces anxiety and depression in HD patients	[69]
	Male Wistar rats	–	A combination of silymarin, quercetin, and hesperidin is more effective than monotherapy in restoring learning and memory loss due to HD	[70]
	Rats	25, 50, and 100 mg/kg	Sesamol and quercetin can be effective in managing HD	[71]
Multiple sclerosis	Mice	150 and 300 mg/kg	Quercetin nanophytosomes are recommended for their potential to improve inflammation and reduce it in patients with MS	[72]
	Th17 cells	100 μm	Quercetin penta acetate has more effective immunomodulatory effects on Th17 cells of MS patients compared to quercetin alone	[73]
Spinal cord injury	Female Sprague–Dawley rats	100 mg/kg	Reduced tissue damage and enhanced neurological function recovery	[74]
	Male Sprague–Dawley rats	7.5 mg/kg	Inhibited necroptosis of oligodendria and suppressed the immune response mediated by M1 macrophages/microglia after SCI	[75]
	Sprague–Dawley rats	20 mg/kg	Decreased neural tissue damage and enhanced astrocyte activation in rats after SCI	[76]
	Sprague–Dawley rats	0.2 mg/kg	Quercetin has protective effects and mechanisms of action in acute SCI	[77]
	Male Sprague–Dawley rats	–	Resveratrol and quercetin prevent secondary damage in SCI	[78]
	Rats	20 mg/kg	Combated SCI‐induced oxidative damage	[79]
	Male Wistar rats	25 μmol/kg	Reduced inflammation after SCI	[80]
	Male Wistar rats	25 μmol/kg	Quercetin administration after SCI in a rat model significantly restores motor function and correlates with motor function recovery in the same model	[81]
	Sprague–Dawley rats	200 mg/kg	Decreased lipid peroxidation levels after SCI	[82]
	Male Wistar rats	25 μmol/kg	Altered S‐100β levels in SCI	[83]
	Sprague–Dawley rats	–	The combination of quercetin and hyperbaric oxygen therapy effectively reduces the progression of damage in a traumatic SCI	[84]
	Rats	25, 50, and 100 mg/kg	Improved motoneuron survival and axonal regeneration	[85]
	Rats	20 mg/kg	Reduced the excitotoxicity of spinal cord motoneurons in rats	[86]
Others	Mice	30 mg/kg	Combated LPS‐induced neurotoxicity in adult mice	[87]
	SH‐SY5Y cells	–	Quercetin has the potential to prevent NDs caused by OS and apoptosis	[88]
	Rats	25 mg/kg	The combination of fish oil quercetin showed neuroprotective effects against OS induced by 3‐NPA in the rat brain.	[89]
	Mice	30 mg/kg	Decreased neuroinflammation and enhanced memory and cognitive function	[90]
	Rats	25 mg/kg	Altered molecular targets are involved in brain cholinergic signaling and reduced cadmium neurotoxicity	[91]
	Wistar rats	50 mg/kg	Quercetin exhibits a neuroprotective effect on locomotor activities and cholinergic neurotransmission in rats	[92]
	Mice	20 and 40 mg/kg	Quercetin has neuroprotective properties that can be utilized for stress treatment and management	[93]
	SH‐SY5Y cells	–	Protected against oxidative damage, potentially aiding in treating oxidative‐related diseases like neurodegeneration	[94]
	Rats	20 and 100 mg/kg	Quercetin can slow down the progression of autoimmune epilepsy and stimulate neuronal recovery in injured parts	[95]
	Rats	1, 5, and 15 μM	Impacted the expression of the *Nrf2* gene	[96]
	Rats	50 mg/kg	Affected OS in a rat of demyelination	[97]
	Oligodendrocyte precursor cells	25 or 50 mg/kg	Enhanced optic pathway repair by protecting the myelin sheath and reducing glial activation	[98]
	Rats	50 mg/kg	Inhibited neuronal autophagy and apoptosis in a rat TBI	[99]
	Male Wistar‐Albino rats	50 mg/kg	Quercetin exhibits neuroprotective properties in radiation‐induced brain injury	[100]

Abbreviations: 3‐NPA, 3‐nitropropionic acid; 6‐OHDA, 6‐hydroxydopamine; AD, Alzheimer's disease; HD, Huntington's disease; MS, multiple sclerosis; LPS, lipopolysaccharides OS, oxidative stress; PD, Parkinson's disease; SCI, spinal cord injury; TBI, traumatic brain injury.

### 4.1 Quercetin and AD

**FIGURE 1:**
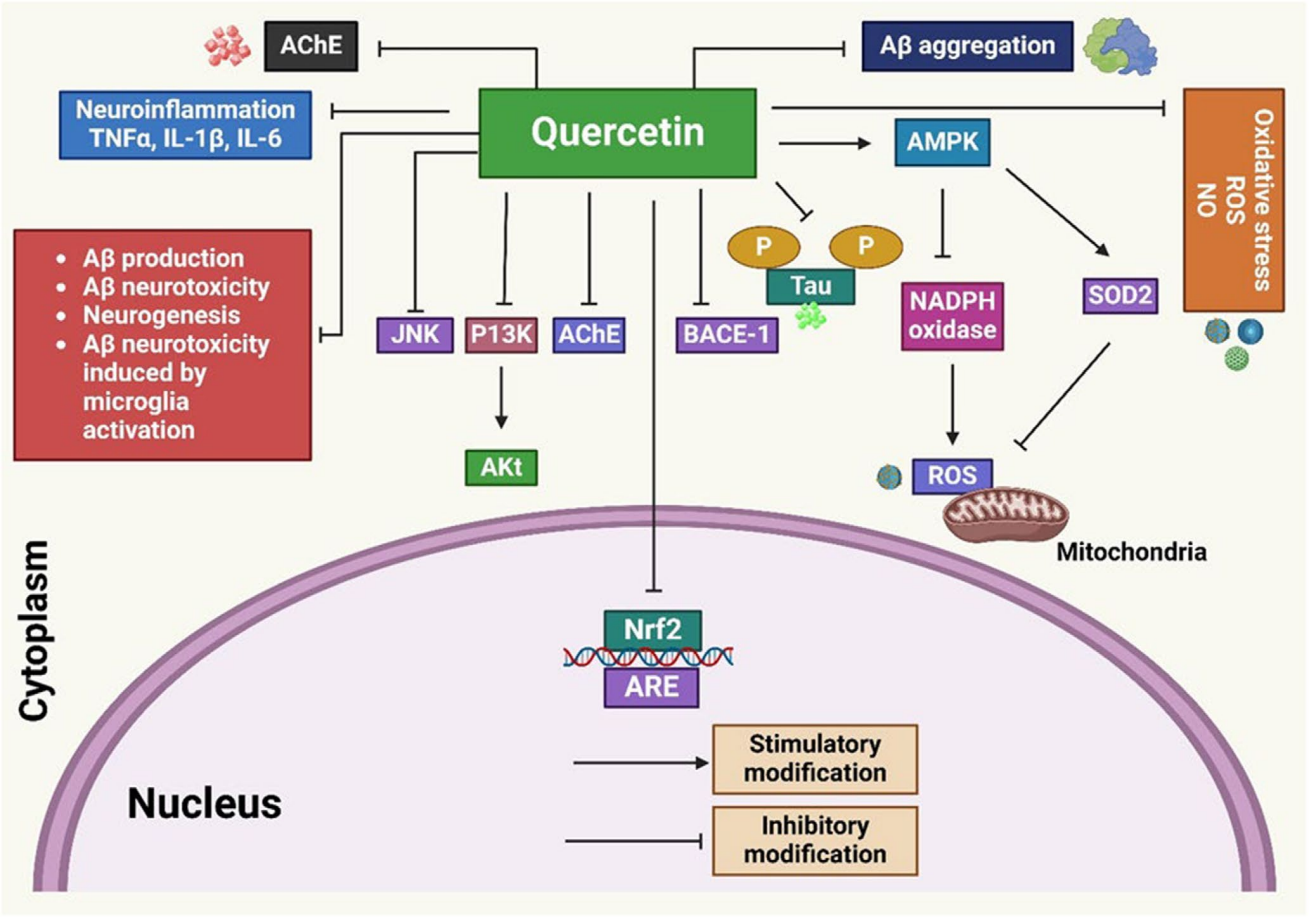
Quercetin exhibits neuroprotective effects in AD (Alzheimer's disease). It affects pathways such as JNK (c‐Jun N‐terminal kinase), PI3K/Akt, AChE (acetylcholinesterase), *Nrf‐2*, BACE‐1 (beta‐site amyloid precursor protein cleaving enzyme), and tau protein hyperphosphorylation.

A critical characteristic of AD is the accumulation of Aβ.^101^ Quercetin has demonstrated therapeutic efficacy in enhancing learning and memory in AD (Figure 1).^102^ Quercetin administration inhibited the enzymes secretase and AChE, which prevented acetylcholine degradion and reduced the Aβ production, respectively.^103^, ^104^ In the amygdala and hippocampal region, quercetin treatment reverses the accumulation of amyloid proteins outside cells and reduces tauopathy, microgliosis, and astrogliosis.^42^ Quercetin maintains mental and sensitive performance in age‐triple transgenic AD animals by inhibiting fibril Aβ protein development and blocking inflammatory cascades.^105^, ^106^ Additionally, quercetin inhibits amyloid precursor protein (APP) maturation, which modifies Aβ production and aggregation.^107^ Quercetin suppresses inducible nitric oxide synthase (iNOS) and regulates cyclooxygenase‐2 (COX‐2) expression in various animal models. Its methylated, sulfated, and glucuronidated metabolites are highly absorbed and exhibit neuroprotective properties.^108^ The study showed the AChE level evaluates memory activity and cholinergic system activity. Quercetin significantly reduced the concentration of AChE in the hippocampus neuronal homogenate and improved the cognitive output of the animals.^53^ In addition, quercetin protects against Aβ (1–42) induced oxidative cell toxicity in cultured neurons.^42^ The antioxidant properties of quercetin were demonstrated to be concentration dependent through in vitro research.^109^ Moreover, it suppresses iNOS and controls COX‐2 expression in various animals.^42^ Additionally, quercetin modifies Aβ production and aggregation by reducing APP maturation.^107^ Quercetin glycosides exhibit neuroprotective effects through opposing gene expression changes.^56^ Furthermore, quercetin has reduced β‐amyloidosis, microgliosis, astrogliosis, and tauopathies.^40^, ^42^ A study on quercetin and its glucosides found that repeated administration before hypoxic–ischemic stroke prevents neuron loss in the striatum and dorsal hippocampus. Quercetin and its glucosides were injected intraperitoneally or intravenously due to their limited bioavailability for neuroprotective effects.^110^ Another study found a correlation between quercetin's ability to increase cerebral blood flow and energy metabolism and its capability to improve memory in mice.^111^ The bioavailability of quercetin in humans is influenced by its significant metabolism during the absorption process of the gut after oral ingestion. It has a limited BBB penetrability.^112^ OS is decreased due to its inhibition of reactive oxygen species (ROS) and reactive nitrogen species (RNS) production and activation. Additionally, quercetin exhibits neuroprotective effects in various NDs and suppresses mitochondrial dysfunction.^113^, ^114^ Aβ aggregation/accumulation and Aβ‐induced neurotoxicity are further enhanced by quercetin, which reduces the amount of ROS.^115^ Quercetin can inhibit the AChE enzyme and decrease ACh metabolism in presynaptic receptors and synapses, improving cognitive functioning.^113^, ^116^ Moreover, Aβ accumulation may impair calcium homeostasis, resulting in the death of neural cells and mitochondrial malfunction.^102^ Quercetin demonstrated antioxidant properties by preventing H_2_O_2_‐induced neuronal damage. It reduced mitochondrial damage and Aβ‐induced neurodegeneration. It also decreased the production of ROS.^117^ Quercetin reduced the death of cells produced by chemical anoxia and H_2_O_2_. It suppressed ROS in rat glioma C6 cells.^118^ In addition, quercetin decreased inflammatory markers, which prevented damage to neuronal cells.^119^ Quercetin exhibited antioxidant activity by reducing malondialdehyde (MDA) levels and enhancing catalase (CAT), superoxide peroxide X (SPX), and superoxide dismutase (SOD) levels in the frontal brains of rats. It reduced neuronal cell degeneration in the frontal cortical cerebral area.^120^ Furthermore, quercetin decreased Aβ levels in an aged AD mouse model, reducing microgliosis, astrogliosis, and amyloidosis in the amygdala and hippocampus. After quercetin injection, the mouse model demonstrated enhanced cognitive and psychological function.^42^

### 4.2 Quercetin and PD

**FIGURE 2:**
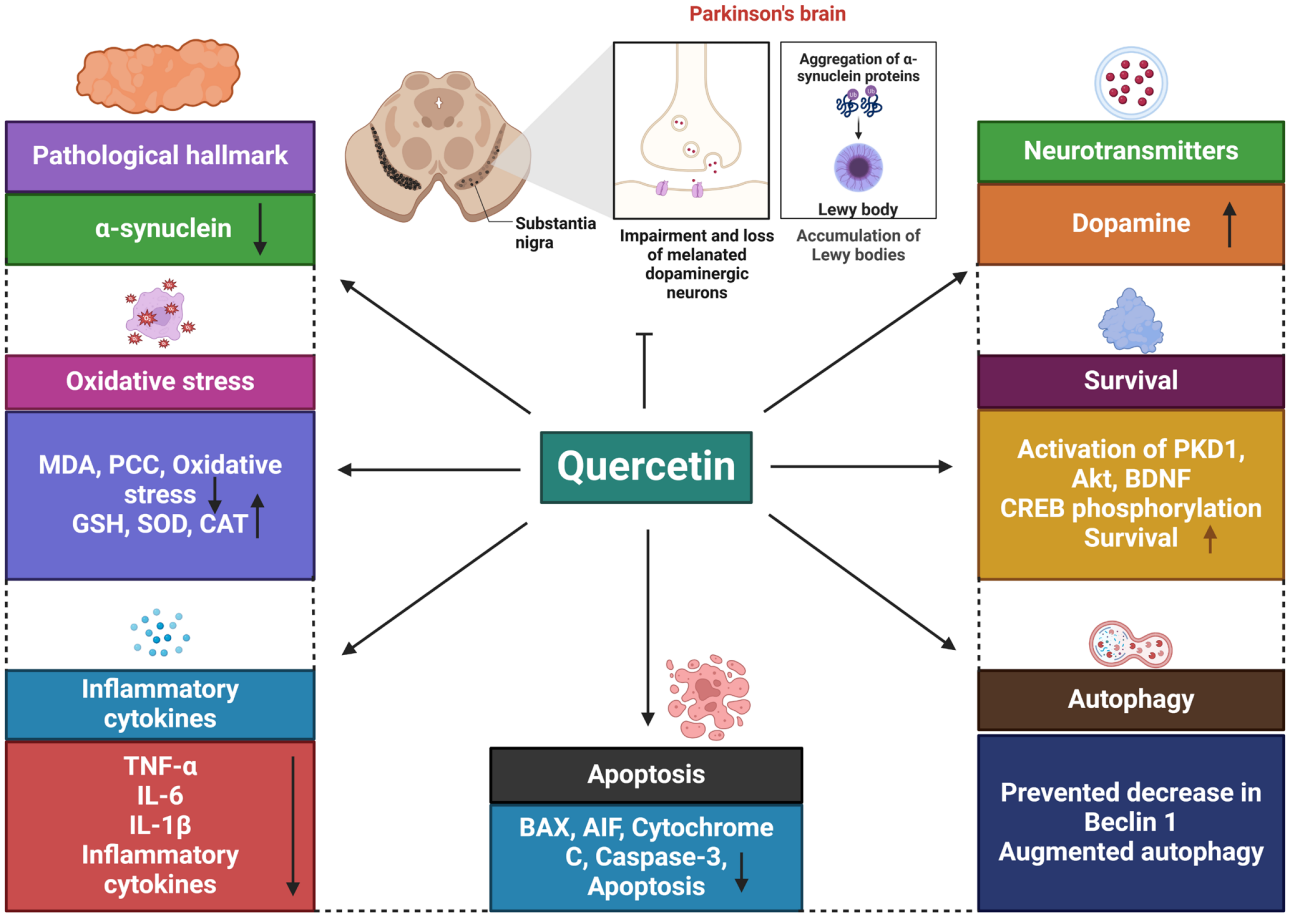
It demonstrates the neuroprotective effects of quercetin in PD (Parkinson's disease) by focusing on its molecular and cellular regulation.

PD is characterized by symptoms such as bradykinesia and shock.^121^ Isoquercetin was found to enhance motor abilities and decrease α‐synuclein fibrillization in PD. In transgenic mouse models of PD, quercetin prevents mitochondrial dysfunction and the gradual degradation of dopaminergic neurons.^114^ The cholinergic deficit is linked to cognitive decline in PD, and quercetin treatment can improve the cognitive decline caused by 6‐OHDA administration.^122^ Oral quercetin administration has a positive effect by decreasing striatal dopamine loss and increasing OS markers.^123^ Oral quercetin treatment can moderately reduce behavioral impairments, nigrostriatal degeneration, and striatal dopamine loss. Quercetin glycoside rutin improves motor impairments.^124^ A study found quercetin increases brain glutathione (GSH). and dopamine levels, inhibits pro‐inflammatory genes in zebrafish, and prevents excessive iNOS and NO production in PC12 cells and zebrafish models.^65^ Another study found that 5‐hydroxytryptamine (5‐HT) depletion in the PD group was reversed, whereas dopamine levels remained unchanged. The dopamine levels and oxidative balance were restored in rats with rotenone‐induced PD, enhancing mobility after intraperitoneal administration of quercetin.^54^ Quercetin at higher doses increased antioxidant enzyme supply and dopamine and ACh levels in a PD experiment with MPTP‐induced PD. The striatum showed a decrease in peroxidation products, specifically 4‐Hydroxynonenal (4‐HNE).^58^ A study showed the impact of quercetin on cognitive abilities in rats with PD. Male Wistar rats were administered different dosages of quercetin, and their spatial memory was assessed. Quercetin enhanced spatial memory by reducing oxidative damage and increasing neuron density.^53^ Moreover, another study explored quercetin's neuroprotective properties in vitro using 6‐OHDA‐treated PC12 cells and in vivo using a PD rat model. Quercetin treatment enhanced mitochondrial quality control–diminished OS, and increased Parkin and Pink1 levels in mitophagy markers. It improved neuronal death and mitochondrial injury in PD rats, reducing progressive motor abnormalities caused by 6‐OHDA. Its neuroprotective impact was reduced by the reduction in Pink1 or Parkin.^55^ Quercetin has exhibited neuroprotective effects by increasing dopamine levels in the brain's extrapyramidal system and reducing α‐synuclein, MPTP, OS, neuroinflammation, and apoptosis levels.^125^ The combined impacts of quercetin and piperine against MPTP‐induced PD in rats were reported.^126^, ^127^ Quercetin and piperine recovered strength, motor coordination, locomotor activities, and body weight from MPTP when administered alone. Together, these reduce inflammatory cytokines.^127^ A study showed the neuroprotective properties of quercetin against apoptosis in the hippocampal region of Wistar rat brains and neurotoxicity caused by aluminum. It decreases aluminum‐induced OS by increasing mitochondrial SOD activity and decreasing ROS levels. It also decreases aluminum's effects on cyt‐c translocation, Bcl‐2 upregulation, Bax downregulation, p53 activation, caspase‐3 activation, and decreased DNA fragmentation.^128^ Quercetin is used to prevent and treat PD (Figure 2).

### 4.3 Quercetin and HD

**FIGURE 3:**
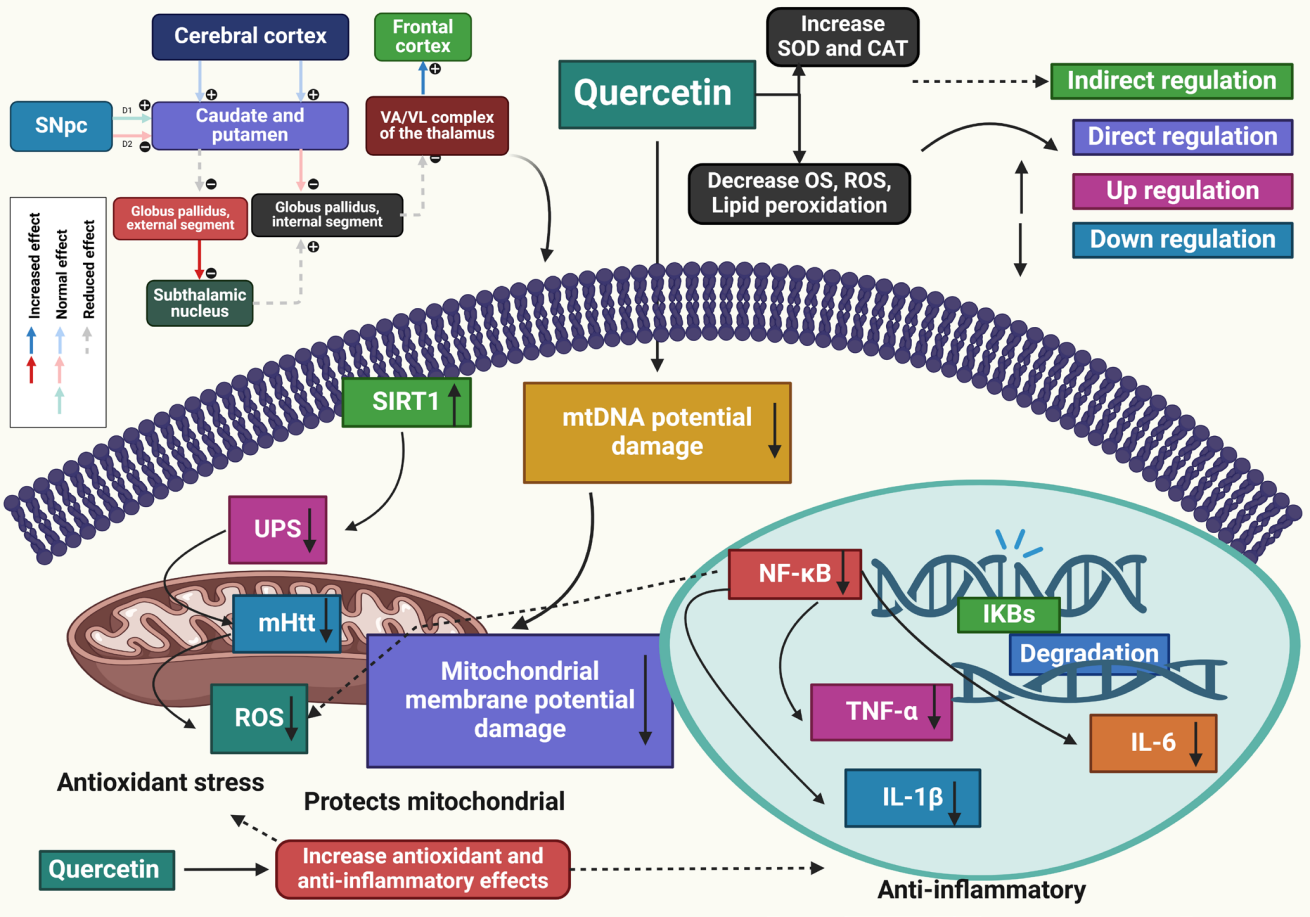
Quercetin reduces HD (Huntington's disease) by targeting SIRT1 (sirtuin 1) to release mHTT (mutant huntingtin) aggregation, restore mitochondrial function, and decrease inflammation.

HD causes neuropathological changes.^129^ A study investigated the advantages of supplementing with quercetin for HD. Quercetin supplementation can inhibit respiratory chain reactions, replenish ATP, reduce mitochondrial OS, and prevent mitochondrial enlargement posttherapy. The study investigated changes in tissue structure, mitochondrial expansion, cellular energy production, oxygen‐induced cell damage, and neurological impairments. Quercetin treatment enhances catalase and SOD activities. It exhibits positive effects on motor defects. Histopathological studies showed HD mice had striatum astrogliosis and pyknotic nuclei, which were absent or significantly reduced in animals treated with quercetin. Quercetin injection reduces OS, mitochondrial dysfunctions, and neurobehavioral impairments, suggesting its potential in managing HD and preserving mitochondrial functions.^130^ Another study investigation assessed quercetin's protection against neurotoxicity caused by quinolinic acid (QA). QA significantly decreased norepinephrine, serotonin, and dopamine levels in the rat forebrain, increasing TNF‐α levels and indicating neuroinflammatory injury.^71^ Additionally, a study found quercetin reduced motor impairments, as measured using footprint analysis and narrow beam walk test. Moreover, 3‐NPA's molecular alterations were reversed, leading to decreased ATP concentration, increased OS, and inhibition of the mitochondrial respiratory chain complex.^130^ Furthermore, the 3‐NPA‐induced changes in the striatum were either mitigated or not present. However, this study^131^ could not substantiate quercetin's protective role against striatal neuronal damage caused by 3‐NPA. In addition, the rats were male, and the intraperitoneal treatment of 3‐NPA and quercetin lasted for only 4 days. Furthermore, the 50‐mg/kg dose of quercetin alleviated additional symptoms such as anxiety, motor impairment, and weight loss. The study found that combined lycopene and quercetin can protect against HD symptoms in rats while reducing locomotor activity without affecting body weight.^69^ Another study investigated the benefits of quercetin supplementation in a HD model. Rats were administered 3‐NPA for 17 days and then quercetin orally for 21 days. The results showed that quercetin counteracted the inhibition of respiratory chain complexes, increased ATP levels, reduced OS, and stopped mitochondrial swelling. It restored thiol content and catalase activity. It also preserved mitochondrial functions and managed HD (Figure 3).^130^ The combined therapy of lycopene and quercetin, with or without poloxamer 188, has been found to effectively reduce anxiety and depression in HD patients.^69^ To cure HD in rats, the study showed the impacts of quercetin on mitochondrial dysfunction caused by 3‐NPA. 3‐NPA causes OS, neuroinflammation, mitochondrial malfunction, and impaired motor control, among other neurotoxic consequences in the brain. Quercetin decreases mitochondrial edema and OS by reducing the respiratory chain's reaction cascade and increasing ATP levels. By increasing the activities of SOD and catalase, the oral dose of quercetin demonstrated antioxidant benefits. Additionally, administering quercetin orally increased motor dysfunction, as determined by analyzing narrow beam walking in footprints. The study found that the striatum of 3‐NPA‐induced groups had more irregularly injured cells with reduced and pyknotic nuclei. Quercetin reversed the 3‐NPA‐induced neurodegenerative alterations.^130^

### 4.4 Quercetin and multiple sclerosis

**FIGURE 4:**
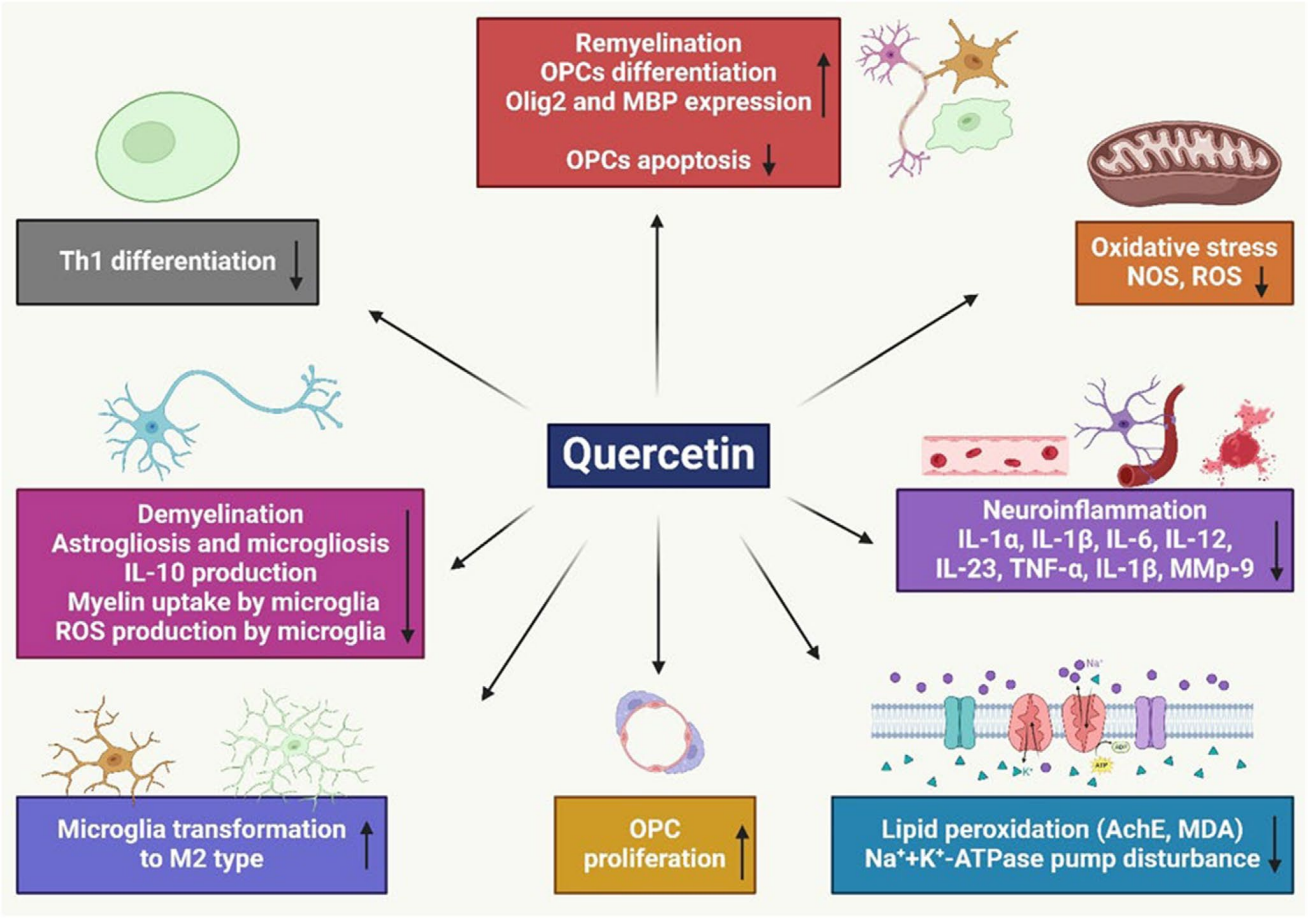
It explores the potential neuroprotective effects of quercetin to prevent MS.

Quercetin penta acetate may be able to take part in clinical trials or be used as a supplement with other MS drugs due to evidence that it has more substantial immunomodulatory properties than quercetin when it comes to Th17 cells of MS patients.^73^ A study showed the role of quercetin in regulating the immune response of peripheral blood mononuclear cells (PBMC) in MS and healthy patients. It inhibited PBMC proliferation, adjusted TNF‐α and interleukin‐1 beta (IL‐1β) levels, and decreased matrix metalloproteinase‐9 (MMP‐9) synthesis. Quercetin and interferon beta (IFN‐β) exhibited additive effects on TNF‐α and MMP‐9 modulation.^132^ Another study evaluated the therapeutic effects of quercetin nanophytosome on inflammatory markers in MS. Quercetin and its nanophysiome significantly reduced inflammatory markers while increasing IL‐10 levels. These can effectively reduce inflammation in MS patients.^72^ Additionally, a study found 156 genes regulated by quercetin, demonstrating potential therapeutic benefits for MS. Animal studies showed quercetin reduces inflammation in the central nervous system (CNS) and onset time in MS models. This quercetin's clinical indications confirm its therapeutic impact on MS.^133^ Quercetin therapy significantly enhanced the outcomes of experimental autoimmune encephalomyelitis (EAE) rats compared to those without any medication. EAE animals exhibited significantly higher brain tissues, myeloperoxidase activity, and serum nitric oxide levels than normal control rats. The study found that rats affected by EAE had lower serum uric acid levels than normal control rats. Quercetin is a potentially beneficial addition to a patient's MS treatment regimen (Figure 4).^134^

### 4.5 Quercetin and ALS

**FIGURE 5:**
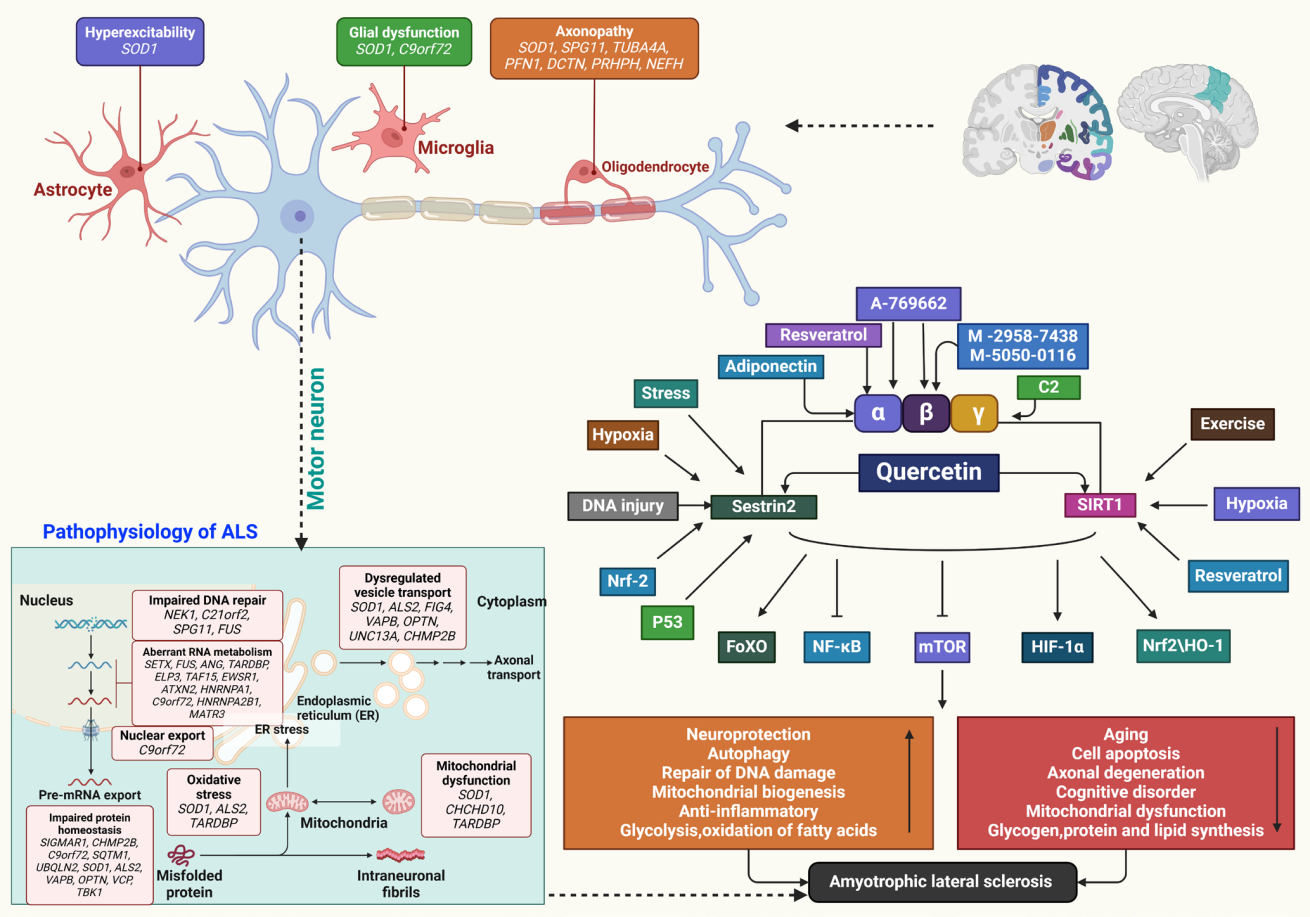
Quercetin has neuroprotective properties. It activates the Sestrin2‐AMPK‐SIRT1 (sirtuin 1) axis for improving ALS.

ALS is a severe ND affecting the CNS. The diagnosis of ALS is becoming easier due to the recognition of its phenotypic heterogeneity. The prognosis of ALS is developing due to discoveries, biomarker identification, predictive models, and clinical trial pipelines for mechanism‐based therapeutics.^135^ Quercetin exhibits biological properties such as neuroprotective, antioxidation, and anti‐inflammatory effects. The study investigated the interaction between quercetin and the AMPK‐SIRT1 (sirtuin 1) axis in NDs. Quercetin stimulates the Sestrin2/AMPK/SIRT1 pathway, improving ALS (Figure 5).^136^ Motor neurons in ALS frequently exhibit misfolding and aggregation of mutants. Researchers discovered that presymptomatic mice and patient tissue contain homodimeric SOD1 monomers, suggesting monomerization may be the initial step in pathogenic SOD1 misfolding. They docked 4400 chemicals to two locations near the SOD1 dimer interface and found quercitrin, quercetin‐3‐*β*‐d‐glucoside, and epigallocatechin gallate (EGCG) to counteract hydrogen peroxide–induced misfolding and aggregation.^137^ Quercetin is significant in ALS therapy by reducing the levels of many anti‐ALS indicators, including ROS, MDA, and SOD1.^137^ Its antineuroinflammatory properties and ability to reduce IL‐6, IL‐8, IL‐1β, TNF‐α, and NF‐κB levels have also been documented.^138^ A study demonstrated quercetin's effects on ALS, but other studies revealed quercetin's inhibition of potential ALS biomarkers. Research indicated the protective effects of quercetin against ALS in rats produced by α‐amino‐3‐hydroxy‐5‐methyl‐4‐isoxazolepropionic acid (AMPA). Chronic excitotoxicity and spinal motor neuron degeneration caused by AMPA further induce neuronal death. Additionally, AMPA increased SIRT1 level. Quercetin prevented the degeneration of motor neurons by inhibiting SIRT1 and mitigating the effects of AMPA.^139^ Furthermore, quercetin and baicalein have antiamyloidogenic properties and reduced SOD1 cytotoxicity in both in vitro and in silico studies.^140^

### 4.6 Quercetin and SCI

**FIGURE 6:**
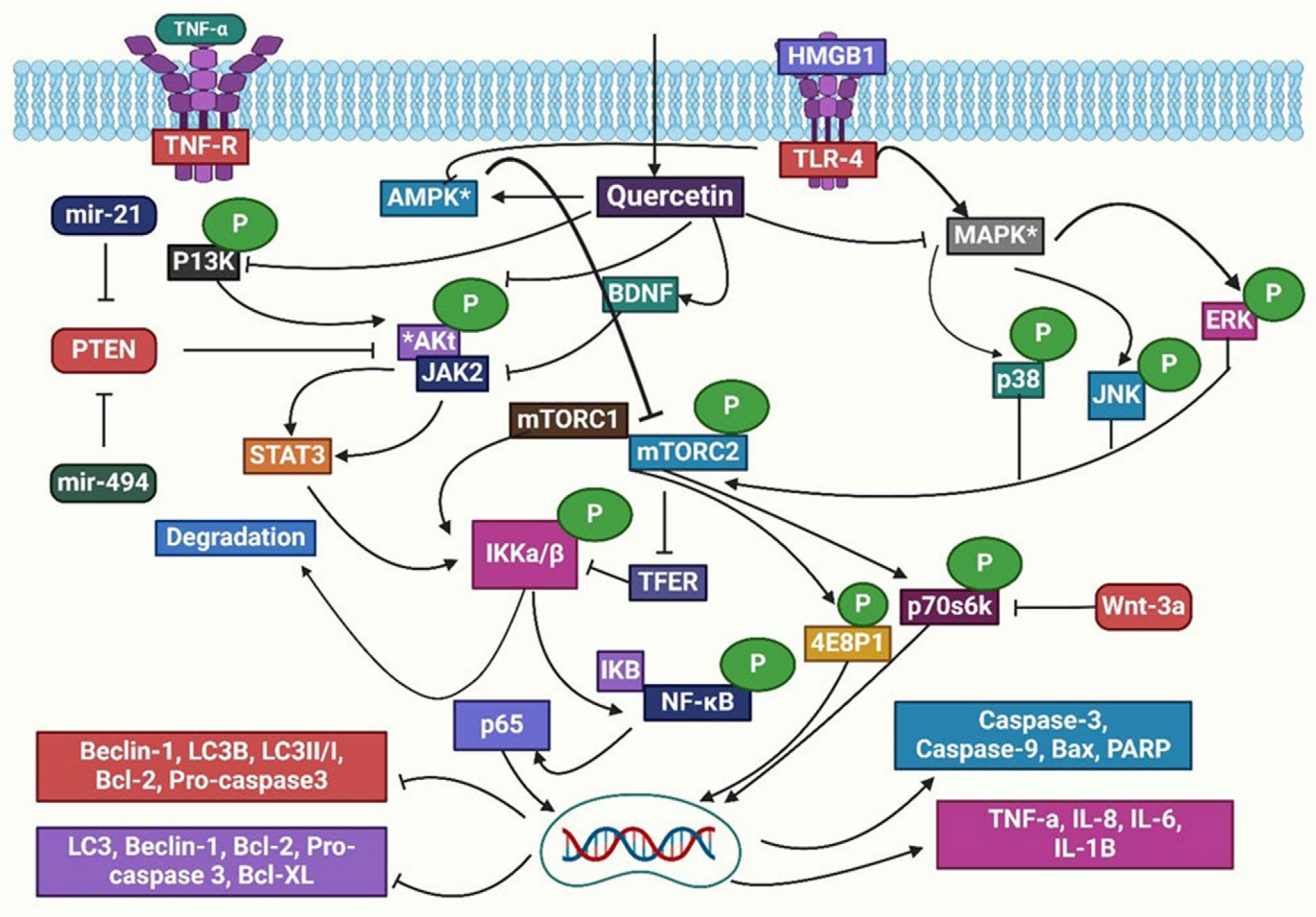
Quercetin exhibits neuroprotective effects on SCI (spinal cord injury). It reduces mTOR activation, PI3K, and Akt phosphorylation, and promotes autophagy by activating AMPK.

SCI increased pro‐inflammatory cytokine production and NOD‐like receptor family pyrin domain containing 3 (NLRP3) inflammasome activation. Quercetin injection reduced ROS formation, inhibited NLRP3 inflammasome activation, and reduced inflammatory cytokine levels. Additionally, administering quercetin reduced histopathology and aided in the return of motility. Quercetin reduces tissue damage and enhances neurological function recovery, which may be due to its inhibition of NLRP3 inflammasome activation.^74^ Quercetin increased functional recovery in rats after SCI. It decreased oligodendrocytes (OLs)' necroptosis without affecting apoptosis or regeneration. It could also be an effective therapeutic target for SCI in clinical settings.^75^ A study found quercetin in SCI‐related neuron regeneration, cavity formation, astrocyte activation, and functional recovery in rats. It aided in astrocyte activation, axonal regeneration, cavity formation reduction, and promotion of locomotor function. It also decreased p‐JNK2 and p‐STAT3 expression and increased BDNF expression.^76^ Quercetin protects the spinal cord of SCI rats by potentially blocking the p38MAPK/iNOS signaling pathway and controlling secondary OS.^77^ In addition, quercetin can enhance antioxidant stress, promote axonal regeneration, and reduce myelin sheath loss, potentially enhancing therapeutic effects in SCI.^141^ Quercetin inhibits neutrophil recruitment to damaged sites, potentially due to its neuroprotective effect, which reduces myeloperoxidase (MPO) release in wounded tissue.^80^ Furthermore, quercetin improves locomotor function and axonal regeneration after SCI. It aids the energy metabolism of SCI and inhibits Akt, mammalian target of rapamycin (mTOR), and p70S6K phosphorylation.^142^ Quercetin is used to prevent and treat SCI (Figure 6).

## 5 SIGNALING PATHWAYS TARGETED BY QUERCETIN

### 5.1 PI3K pathway

Age‐related NDs and severe other brain diseases are linked to phosphoinositide 3‐kinase (PI3K). In the PI3K/Akt pathway, Akt is a regulatory protein that controls the survival and plasticity of neurons. G‐protein‐coupled receptors, integrins, B‐ and T‐cell receptors, cytokines, receptor tyrosine kinase (RTK), and other stimuli can all be involved in the activation of Phosphatidylinositol‐3,4,5‐trisphosphate (PIP3). This causes Akt to become phosphorylated, influencing how different target proteins activate or decrease their function. As a result, it regulates a wide range of cell processes, the most notable of which are the cell cycle, growth, metabolism, proliferation, apoptosis, and protein synthesis.^143^ When given a high‐fat diet, animal models of NDs exhibited decreased learning and memory, which can be directly linked to OS.^144^ The levels of several hippocampus genes, including PI3K/Akt and *Nrf2*, are decreased by this diet high in saturated fats and by the participation of carbonyls and ROS. Quercetin reduces memory and learning loss and increases antioxidant efficiency when combined with high‐fat diet (HFD). Quercetin therapy in ischemic rats improves antiapoptotic and antioxidant signaling and reduces OS, glutathione peroxidase, glutathione, cytochrome c, and lipid peroxidation in the cortex and striatum.^145^ Additionally, the impact of combining quercetin and exercise was eliminated by LY294002 (a PI3K/Akt inhibitor), and the Bax to Bcl‐2 ratio increased. This recommended that the PI3K/Akt pathway was involved in modifying the antioxidant capabilities of both treatments. Quercetin therapy effectively inhibited OS‐induced tau protein phosphorylation, a significant target of tau antihyperphosphorylation, indicating its potential neuroprotective benefits in non‐NDs like AD and PD. Quercetin effectively protected PC12 pheochromocytoma cells from hydrogen peroxide–induced apoptosis by reducing ROS, lactate dehydrogenase, and malondialdehyde levels and increasing glutathione and SOD activities.^146^

### 5.2 Sirtuin pathway

Sirtuins are a significant area in the neuroprotective processes of quercetin. The regulation of metabolism, longevity, and stress responses is a molecular process influenced by sirtuins (seven in mammals, SIRT1 to SIRT7, NAD + ‐dependent deacetylases). SIRT1 is primarily nuclear and is highly expressed in several brain areas, notably the hypothalamus, which is linked to longevity. Calorie restriction diets have been shown to induce SIRT1, exhibit protective effects against dopaminergic neuron neurodegeneration,^147^, ^148^ and reduce Aβ peptide development in chinese hamster ovary cells (CHO) cells and neuronal Tg2576 cultures that expressed APP swedish mutation (APPsw).^149^ SIRT1's multimodal activation regulates proapoptotic transcription factors, Bax‐dependent apoptosis, and Aβ peptide inhibition. The neuroprotective and antiaging activities of quercetin have been linked to the stimulation and activation of stress‐related processes regulated by SIRT1.^150^ A study found that quercetin's activation of these SIRT1‐dependent signaling pathways modulates the release and functioning of inflammatory cytokines, which reduces neuronal demyelination and suggests quercetin for treating MS and ALS.^151^ The actions of quercetin on the SIRT1 pathway were found to cause neurotoxicity in herpes simplex virus type 1 through similar outcomes.^152^

### 5.3 The Nrf2‐ARE pathway

*Nrf2* primarily regulates stress caused by free radicals. The protein Keap1, linked to cullin 3‐base E3 ligase, restores *Nrf2* to the cytoplasm, leading to its ubiquitination and subsequent proteasomal degradation.^153^, ^154^, ^155^ To prevent oxidative damage and cell death, neuroprotection is provided via stimulating the Nrf2‐ antioxidant response element (ARE) signaling pathway. Furthermore, the Nrf2‐ARE pathway can control the formation of misfolded protein aggregates, which are found in various NDs, including AD, PD, and HD.^156^ Additionally, glutathione cysteine synthase (GCS) expression, an essential enzyme in the production of the antioxidant glutathione, is induced by Nrf2‐ARE activation. Through the Nrf2‐ARE signaling pathway, quercetin has demonstrated a propensity to counteract OS‐induced cellular damage.^156^, ^157^ Tertiary butylhydroquinone, a classic *Nrf2* inducer, demonstrated how activation of Nrf2‐ARE protects against Aβ‐induced neurotoxicity,^153^, ^154^ and di‐hydroquercetin.^155^ Quercetin can operate like a neuro‐hormetic phytochemical and activate the *Nrf2* pathway by involving the c‐Jun N‐terminal kinase (JNK) and extracellular signal‐regulated kinase (ERK) signaling pathways.^157^ In addition, quercetin enhances cellular resilience to OS caused by neurotoxic compounds like Aβ peptide or oxidants like hydroperoxide.^9^ Quercetin protects neurons in AD by blocking *Nrf2* ubiquitination, increasing Nrf2 transcription. In general, there is an increase in the *Nrf2* to Keap1 ratio, which increases nuclear *Nrf2* concentrations and intensifies contact with the ARE.^158^ Furthermore, in an animal model of d‐galactose‐induced neurotoxicity, quercetin prevented hippocampus apoptosis and increased *Nrf2* activity, which improved learning and memory.^159^

### 5.4 TNF‐α pathway

TNF, produced by macrophages and monocytes during acute inflammation, is an inflammatory cytokine that triggers several cell signaling pathways, resulting in necrosis and apoptosis. There are two types of TNF molecules: TNF‐α and TNF‐β.^160^ Numerous research studies conducted in vitro and in vivo have proved quercetin's anti‐inflammatory properties. By blocking the TNF‐α pathway, quercetin reduces inflammation caused by 6‐OHDA toxicity.^161^ Additionally, quercetin suppressed microglial‐mediated neuronal cell death in microglia PC12 co‐culture^162^ and the LPS‐induced mRNA expression of TNF and IL‐1 in glial cells and astrocytes.^163^ In human neuroblastoma SH‐SY5Y cells, quercetin‐loaded nanoparticles effectively reduce inflammation by regulating toll‐like receptor 4 (TLR4) and blocking the TNF‐α pathway in response to oxysterol‐mediated stress.^164^ Furthermore, microglial cells are essential in triggering neuronal cell death mediated by an inflammatory cascade by producing neurotoxic and inflammatory mediators like IL‐6, TNF‐α, IL‐1, and NO. Research on quercetin in astrocyte cells has shown that it protects rat glioma cells (C6 cells) from H_2_O_2_ toxicity, which lowers the number of apoptotic cells.^165^ This is achieved by reducing the production of ROS. Rat oligodendrocytes, or OLN‐93, are essential for neurons, and quercetin increases lifespan.^166^

### 5.5 JNK pathway

One of the main signals in the downstream signaling pathway of mitogen‐activated protein kinase (MAPK) is the JNK. JNK is a member of the family of threonine protein kinases, which consists of 3 genes (*JNK1*, *JNK2*, and *JNK3*) that collectively encode 10 different forms.^167^ Whereas *JNK3* is primarily found in the CNS and has therapeutic potential for NDs and other CNS diseases, *JNK1* and *JNK2* are extensively dispersed in many tissues and have a significant contribution to insulin resistance caused by overweight.^168^, ^169^ JNKs are activated by the phosphorylation of threonine and tyrosine residues, and they become inactivated via a negative feedback loop initiated by MAPK phosphatases.^170^ Quercetin reduces the formation of ROS in rats by reducing the expression of JNK and its phosphorylated form and blocking transcription factor activator protein ‐1 (AP‐1) activation. It prevents H_2_O_2_‐induced apoptosis in mesangial cells by AP‐1 pathway activation.^171^ Furthermore, quercetin pretreatment attenuates ERK and JNK kinase activation, which is triggered by the fast phosphorylation of hydrogen peroxide. In many different types of cells, apoptosis‐induced cell death has been reported as being mediated by JNK and its substrate, c‐Jun.^172^ Quercetin reduces JNK activation in RL34 cells caused by 4‐hydroxy‐2‐noneal, the by‐product of lipid peroxidation. It has been suggested that quercetin's inhibitory effects on the HNE‐triggered stress signaling pathways are caused by inhibiting certain enzymes, specifically PKC.^173^ Consequently, quercetin exhibited anti‐inflammatory and protective effects via the JNK signaling pathway, which is investigated in macrophages.^174^

### 5.6 PON2 pathway

PON2 is a gene belonging to the paraoxonase family, which also contains PON1 and PON3.^175^, ^176^ Additionally, PON2 is an intracellular enzyme, whereas PON1 and PON3 are primarily found in the blood and liver.^177^, ^178^ PON2 is predominantly found in the mitochondria.^178^, ^179^ 17‐ β‐Estradiol is found to increase PON2 expression.^180^ In addition, PON2 has antioxidant properties, which are assumed to be essential for neuroprotection and for delaying the atherosclerotic process.^178^, ^179^ PON2 prevents oxidative damage in cells by binding to coenzyme Q10. A deficiency in PON2 results in mitochondrial dysfunction.^179^ Moreover, PON2 selectively and indirectly reduces superoxide emission from the inner mitochondrial membrane without altering the concentrations of hydrogen peroxide and peroxynitrite.^181^ A favorable regulation of PON2 may lead to neuroprotection because of its antioxidant activities in the CNS.^182^ OS increases PON2 expression in macrophages,^183^ and the promoter area of PON2 in vascular cells was shown to have a sequence similar to the endoplasmic reticulum stress element.^184^ It has been demonstrated that a variety of substances increase PON2 expression in a range of tissues or cell types.^185^ A study found the in vitro activation of PON2 in brain cells by quercetin.^186^ Furthermore, PON2 protein expression is elevated by quercetin in macrophages, mouse striatal astrocytes, and neurons. The peroxisome proliferator‐activated receptor (PPAR) γ inhibitor GW9662 does not counteract the effects of quercetin, whereas the JNK/AP‐1 pathway inhibitor SP600125 does. One theory is that quercetin could cause minimal OS.^156^, ^187^

### 5.7 JAK–STAT pathway

Numerous diseases are NDs, such as MS, AD, PD, and other ailments involving the degradation of neurons and/or glia.^188^ Once considered immune privileged, the CNS consists of T cells constantly attacking it, with innate immunity as its primary defense line. The abnormal activation of innate immune cells leads to the release of NO, ROS, chemokines, and pro‐inflammatory cytokines, or polarizes and activates T cells, myeloid cells, and effector T cells, which is the cause of demyelination and/or degeneration of neurons. Many NDs are associated with CNS inflammation, which is caused by the JAK–STAT signaling system and results in pathogenic inflammation. Most of these operations rely on these signaling pathways.^189^ The T‐helper type 1 (Th1) cells control STAT1 signaling and produce cytokines that change the ratio of Th1 to Th2 cells, modifying inflammation and immune response. However, mutations in the STAT1 gene cause chronic *Mucocutaneous candidiasis* and negatively impact Th1 and Th17 cell responses due to STAT1 hyperactivation and poor nuclear dephosphorylation.^190^ Quercetin can treat NDs by reducing neuroinflammation through the JAK–STAT signaling pathway, alone or in combination with other medications. Nanoliposomes can increase quercetin's ability to prevent cancer by blocking the JAK2‐STAT3 pathway and lowering the production of ROS in mitochondria. Furthermore, quercetin becomes more easily soluble across the BBB when combined with nanoparticles like β‐cyclodextrin‐dodecyl carbonate, which makes it a significant option for neuroinflammatory therapy and STAT intervention.^191^

## 6 THROUGH AMPK, QUERCETIN INHIBITS INFLAMMATION AND PROTECTS AGAINST NF‐ΚB AND THE NLRP3 INFLAMMATORY CYTOTOXICITY IN NEUROINFLAMMATORY TOXICITY

Quercetin's neuroprotective and anti‐inflammatory properties are attributed to its modulation of AMPK NF‐κB and NLRP3 inflammasome pathways, which regulate gene expression for cell survival and inflammation. The regulation of pro‐inflammatory cytokines and OS is linked with the NF‐κB signaling system. Quercetin suppresses of NF‐κB activation.^192^, ^193^ Quercetin inhibits the activation of the NLRP3 inflammasome.^194^ The cytosolic complex known as the NLRP3 inflammasome, which is made up of the proteins NLRP3, apoptosis‐associated speck‐like protein containing a CARD (ASC), and caspase‐1, is essential for controlling the immune system's reaction to different types of cellular stress and danger signals.^195^, ^196^ The NLRP3 inflammasome, a group of proteins, controls inflammation and cell death by cleaving pro‐inflammatory cytokines like IL‐18 and IL‐1β. Quercetin is a neuroprotective agent that regulates cellular pathways involved in controlling NF‐κB and the NLRP3 inflammasome during neuroinflammatory toxicity, thereby controlling inflammation and cell death.^197^, ^198^

## 7 IN VITRO AND IN VIVO STUDIES

Quercetin has neuroprotective properties. It prevents neuronal cell toxicity by triggering OS at low micromolar doses. Its metabolites also undergo methylation, or sulfation postabsorption, providing neuroprotection.^199^, ^200^, ^201^ Quercetin counteracts cell toxicity caused by oxidants and neurotoxic molecules.^112^, ^186^, ^202^ In addition, quercetin glycosides, specifically isoquercitrin and rutin, may counteract gene expression changes caused by 6‐OHDA in PC12 cells.^56^ Quercetin decreased OS in secluded rat brain mitochondria and counteracted the toxic effect of the anticancer medication oxaliplatin.^203^ Aβ‐peptide toxicity protects neural cells from damage.^109^ Additionally, quercetin exhibits neuroprotection in vitro at concentrations in the micromolar range.^204^ The majority of quercetin absorbed is found in the metabolites. Certain quercetin metabolites that undergo glucuronidation, methylation, and sulfation exhibit neuroprotective properties in vitro.^21^, ^22^, ^205^ Quercetin can counteract OS and provide neuroprotection. For instance, oral quercetin (0.5–50 mg/kg) has been demonstrated to prevent mice from neurotoxicity and OS brought on by various neurotoxicity.^27^, ^29^ Moreover, quercetin protects against the neurotoxicity of tungsten, lead, and methylmercury.^206^, ^207^ Quercetin reduces the neurotoxicity of polychlorinated biphenyls, the pesticide endosulfan, and MPTP in vivo.^204^, ^208^ Additionally, quercetin counteracts the cognitive decline that mice fed a high‐fat diet produced.^144^ Quercetin proved neuroprotective in rat models of intracerebral hemorrhage^209^ and prevented the retina in a rat model from apoptosis brought on by ischemia–reperfusion injury.^210^ Furthermore, quercetin enhances the progression of AD pathology and cognitive abnormalities in an elderly triple‐transgenic AD mouse model.^42^ A study found that combining quercetin and fish oil oral supplementation increased neuroprotective activity in rats exposed to rotenone or 3‐NPA over an extended period.^62^, ^211^ Quercetin significantly reduced catalepsy and exhibited neuroprotective properties in PD caused by rotenone. It reversed levodopa's (l‐dopa) toxic effects, improving neurochemical parameters and normalizing the rotarod score. Quercetin's potential as a disease‐modifying treatment is indicated by its potent iron‐chelating properties when combined with l‐dopa.^212^ Another study found that isoquercetin enhanced motor functions in cases of acute SCI, decreased α‐synuclein fibrillization, enhanced synaptic plasticity, decreased hippocampal neuronal cell death, and reverted the histopathological characteristics of AD.^213^ Quercetin prevents mitochondrial dysfunction and the gradual degeneration of dopaminergic neurons. The study investigates PD using cell culture and MitoPark transgenic mouse models.^114^ Administration of quercetin in vitro models led to suppressing AChE and secretase enzymes, thereby blocking the breakdown of acetylcholine and reducing Aβ formation, respectively.^104^, ^214^

## 8 NEUROPROTECTIVE EFFECTS OF QUERCETIN IN PRECLINICAL AND CLINICAL TRIALS

Quercetin has been confirmed in scientific studies to possess neuroprotective properties in various animal models of neuronal damage and NDs. There are not many clinical trials utilizing quercetin to investigate its effectiveness for different NDs in people. Quercetin nanoparticles were administered for trials in 3 of 14 studies. To activate regulated quercetin administration in the brain, quercetin nanoparticles functionalized with poly(lactic‐co‐glycolic acid) PLGA were developed.^215^ Two other investigations also showed the effectiveness of quercetin, zein, and lipid nanoparticles in AD models.^41^ Furthermore, quercetin's limited water solubility and limited permeability to the BBB cause poor oral bioavailability, which inhibits the drug's therapeutic application. However, the use of nanoparticles in dosage form administration has shown promise in producing targeted, focused activities. Thus, it was suggested that using quercetin nanoparticles as oral carriers would significantly enhance brain performance and, as a result, improve NDs in both preclinical and clinical settings.^216^ A study analyzed 14 animal studies evaluating the preventive effect of quercetin in AD models from 3 databases. The outcomes of the studies supporting quercetin's anti‐AD effectiveness in preclinical trials were strong.^217^ Regarding quercetin's potential applications in the future, cognitive‐enhancing activities appear to be the most promising. Interestingly, because the substance is generally regarded as safe, positive outcomes of a large number of preclinical and molecular investigations motivated researchers to conduct clinical trials. Based on a previous study from 2010,^218^ which found that administering 2000 mg of quercetin right before the vigilance task caused an upward trend in task efficiency, this study^219^ designed a study to investigate the potential effects of long‐term consumption of quercetin at dosages typically recommended in supplementation on cognitive performance. Quercetin has advanced to phase II of the Clinical Trial to Assess Senolytic Therapy's Safety and Feasibility in Alzheimer's Disease. An experiment in a mouse model of AD demonstrated that the medication combinations used in the trial prevented neurons from death. Dasatinib and quercetin are senolytic substances that specifically remove senescent cells and are associated with various age‐related or age‐predisposed diseases.^220^ When administered together, they have greater effects in vitro than alone. Enrolled in the clinical trial are elderly people who have early‐stage AD (tau‐positive) or minor cognitive deficits. Together with a capsule containing 100 mg of dasatinib, a daily oral dose of 1000 mg of quercetin is administered in four doses. The combination medication is administered twice a row, followed by a 13‐day break without medication, two more days of medication application, and so on until the six administration cycles are completed. Before that experiment, a preliminary investigation known as the senolytic therapy to modulate the progression of Alzheimer's disease (SToMP‐AD) was carried out using cerebrospinal fluid analysis to see whether the medications affected brain penetration.^221^ Preclinical research has shown quercetin to be a highly effective drug against NDs, suggesting a positive outcome for human therapies.^221^ Quercetin was able to exhibit neuroimaging signals in older AD populations, overcome the BBB, and reduce cognitive impairment.^221^ Furthermore, another study showed improvement in depressive symptoms and increased desire, thereby preventing cognitive deterioration.^222^

## 9 CONCLUSION AND FUTURE PERSPECTIVES

Within neuropharmacology, there is growing interest in quercetin's potential as a treatment for NDs. Quercetin has been extensively studied for its neuroprotective properties both in vitro and in vivo. The effects of this substance are mediated through its interaction with various cellular and molecular signaling pathways. Quercetin effectively combats dementia's pathophysiological mechanisms, such as OS, mitochondrial dysfunction, and inflammatory responses, by regulating these pathways. Research on quercetin's therapeutic use in treating NDs such as AD, PD, HD, and MS is limited, with most preclinical studies. Clinical insights from in vitro and in vivo research demonstrate quercetin's ability to target key signaling pathways in NDs. Quercetin's medicinal potency is enhanced by its ability to penetrate the BBB. Despite promising preclinical results, there is still much to learn about applying quercetin's therapeutic benefits in clinical settings. Innovative liposomes, nanoparticles, or conjugations with other compounds could enhance the stability and bioavailability of quercetin, potentially enhancing its therapeutic efficacy. Clinical trials are essential for determining quercetin's therapeutic dosage, safety, and efficacy in humans, with subsequent research focusing on comparative analysis with existing neuroprotective remedies. Further research is needed to understand quercetin's molecular pathways to achieve its effects, using advanced technology and systems biology methodologies to uncover new targets. Understanding the impact of environmental variables and genetic predispositions on quercetin's efficacy can lead to the development of personalized treatment techniques for improved outcomes. Research on the long‐term effects of quercetin supplementation is essential, along with developing monitoring instruments to evaluate treatment effectiveness and neuroprotection over time. Quercetin can significantly impact ND management and treatment through focused research and innovation, potentially changing the direction of ND therapy.

## AUTHOR CONTRIBUTIONS

**Md. Rezaul Islam:** Conceptualization; data curation; formal analysis; investigation; supervision; visualization; writing – original draft; writing – review and editing. **Md. Ibrahim Khalil Al‐Imran:** Data curation; resources; validation; writing – original draft; writing – review and editing. **Mehrukh Zehravi:** Conceptualization; data curation; investigation; writing – original draft; writing – review and editing. **Sherouk Hussein Sweilam:** Data curation; investigation; writing – original draft; writing – review and editing. **Mohammad Rakib Mortuza:** Data curation; formal analysis; visualization; writing – review and editing. **Jeetendra Kumar Gupta:** Data curation; formal analysis; resources; validation; writing – review and editing. **Thukani Sathanantham Shanmugarajan:** Formal analysis; investigation; validation; writing – review and editing. **Kadirvel Devi:** Data curation; formal analysis; investigation; resources; writing – review and editing. **Tanuja Tummala:** Data curation; formal analysis; resources; validation; writing – review and editing. **Mohammed Ali Alshehri:** Formal analysis; validation; visualization; writing – review and editing. **Kalirajan Rajagopal:** Data curation; investigation; resources; visualization; writing – review and editing. **Mohammed Asiri:** Formal analysis; funding acquisition; visualization; writing – review and editing. **Irfan Ahmad:** Funding acquisition; validation; visualization; writing – review and editing. **Talha Bin Emran:** Formal analysis; project administration; supervision; validation; visualization; writing – review and editing.

## FUNDINNG INFORMATION

Not applicable.

## CONFLICT OF INTEREST STATEMENT

The authors declare no competing interests.

## ETHICS STATEMENT

Not applicable.

## Data Availability

Not applicable.
